# Diet in Rheumatoid Arthritis versus Systemic Lupus Erythematosus: Any Differences?

**DOI:** 10.3390/nu13030772

**Published:** 2021-02-27

**Authors:** Alessia Alunno, Francesco Carubbi, Elena Bartoloni, Davide Grassi, Claudio Ferri, Roberto Gerli

**Affiliations:** 1 Rheumatology Unit, Department of Medicine, University of Perugia, Piazzale Menghini 1, 06100 Perugia, Italy; elena.bartolonibocci@unipg.it (E.B.); roberto.gerli@unipg.it (R.G.); 2 Internal Medicine and Nephrology Unit, Department of Life, Health & Environmental Sciences, University of L’Aquila, and Department of Medicine, ASL 1 Avezzano-Sulmona, 67100 L’Aquila, Italy; francescocarubbi@libero.it (F.C.); davide.grassi@univaq.it (D.G.); claudio.ferri@univaq.it (C.F.)

**Keywords:** systemic lupus erythematosus, rheumatoid arthritis, cardiovascular risk, Mediterranean diet, polyunsaturated fatty acids

## Abstract

In recent years, an increasing interest in the influence of diet in rheumatic and musculoskeletal diseases (RMDs) led to the publication of several articles exploring the role of food/nutrients in both the risk of developing these conditions in normal subjects and the natural history of the disease in patients with established RMDs. Diet may be a possible facilitator of RMDs due to both the direct pro-inflammatory properties of some nutrients and the indirect action on insulin resistance, obesity and associated co-morbidities. A consistent body of research has been conducted in rheumatoid arthritis (RA), while studies in systemic lupus erythematosus (SLE) are scarce and have been conducted mainly on experimental models of the disease. This review article aims to outline similarities and differences between RA and SLE based on the existing literature.

## 1. Introduction

The relationship between diet and chronic diseases has been extensively investigated with the so-called “Western-type” diet, characterized by high intake of refined grains, desserts and sweets, processed meat, red meat, condiments, and pizza, which are associated with cardiovascular (CV) disease and mortality risk in large prospective cohort studies [1]. Rheumatoid arthritis (RA) and systemic lupus erythematosus (SLE) are systemic autoimmune diseases, and patients suffering with these conditions have approximately double the risk of atherosclerotic CV disease, stroke, heart failure and atrial fibrillation compared with the general population [2,3]. Recently, specific recommendations for the management of CV risk in inflammatory arthritis have been issued, incorporating the concept that besides conventional CV risk factors, chronic inflammation accounts for this increased CV risk [4]. Since this also applies to SLE, a dual approach has been pursued in patients with RA and SLE. This dual approach is based on targeting conventional CV risk factor reduction and on the reduction of disease activity for the primary and secondary prevention of CV events and mortality in these patients [5,6].

Over the last decades, an increasing number of studies explored the possible impact of nutrients and different dietary patterns on the risk of developing rheumatic and musculoskeletal diseases (RMDs), particularly RA [7,8]. Diet may be a possible facilitator of RMDs due to both the direct pro-inflammatory properties of some nutrients and their indirect action on insulin resistance, obesity and associated co-morbidities [9,10]. Further, the balance of macronutrients and omega-6/omega-3 fatty acids in the daily diet has been described to modulate the expression of inflammatory genes [11].

However, single dietary components may confer only modest benefits. Since individual foods are part of complex dietary patterns, in recent years scores of overall dietary quality have received increasing attention in disease prevention. Dietary pattern scores provide a broader perspective on this matter and a relative measure of the healthfulness of an individual’s diet. Hence, they may provide insights not only on the role of individual nutrients but also their combination.

In addition, diet may also have an effect in worsening/improving RMDs in patients with established diseases, and differences may exist across various conditions [8,12].

Our purpose is to outline similarities and differences between RA and SLE based on the existing literature.

## 2. Diet and Risk of SLE Development

While there are several studies exploring the role of nutrients in the risk of developing RA [7], literature on SLE is scarce (Table 1). The Western type of diet was not associated with a higher risk of developing SLE, and the healthiest prudent pattern—reflecting a diet higher in vegetables, fruit, legumes, fish, tomatoes, poultry, and whole grains—was not associated with a lower risk of developing the disease [13].

As far as the scores of overall dietary quality are concerned, the Nurses’ Health Study (NHS) and NHS II provided important insights on this matter. These studies involved two large prospective cohorts that allowed the exploration of the risk of developing RMDs based on dietary habits, monitored over time with validated instruments [14,15].

### 2.1. Alternative Healthy Eating Index (AHEI-2010) and Its Domains

Although a healthier diet, as defined by a higher adherence to the 2010 alternative healthy eating index (AHEI-2010), was associated with a reduced risk of RA, particularly seropositive, occurring at 55 years of age or younger [14], no association between long-term adherence to the AHEI-2010 and SLE risk was observed [15]. The AHEI-2010 was developed by researchers at the Harvard School of Public Health as an alternative measure of diet quality to identify future risk of diet-related chronic disease [17]. It includes a variety of foods/nutrients and defines fruits, vegetables, whole grains, nuts, long-chain n-3 polyunsaturated fatty acids (LC-PUFAs), and moderate alcohol consumption as healthy items, while sugar-sweetened beverages, red/processed meat, trans fat and sodium intake are classified as unhealthy components. As far as AHEI-2010 subdomains are concerned, a higher score of alcohol (moderate intake) or red meat consumption (lower intake) was significantly associated with a reduced RA risk regardless of body mass index (BMI). Conversely, women in the highest AHEI-2010 tertile of nut/legume intake had a decreased risk of developing SLE compared to those in the lowest tertile, but no other association was observed [15].

The data on alcohol consumption and RA are in line with a recent meta-analysis of prospective studies demonstrating that low to moderate intake is inversely associated with the development of RA in a dose, time and gender-dependent manner [18].

On the contrary, the link between alcohol consumption and SLE risk remains controversial. A meta-analysis conducted in 2008 demonstrated that moderate alcohol intake had a protective effect on the development of SLE [19], and the subsequent studies were in line with the previous literature [20,21,22,23]. However, no genetic association in this regard has been identified [24]. Furthermore, no association for a higher risk in heavy alcohol consumers was reported by other studies [25,26].

Other dietary quality scores have also been explored in relation to the risk of RA or SLE onset in the NHS and NHS II cohorts.

### 2.2. Mediterranean Diet, the Dietary Approach to Stop Hypertension (DASH) and the Empirical Dietary Inflammatory Pattern (EDIP)

The Mediterranean diet is a dietary pattern based on whole or minimally processed foods and a high intake of vegetables, fruits, whole grains, fish and olive oil, with moderate consumption of red meat and wine. The adherence to the Mediterranean diet proved to be effective in preventing metabolic diseases such as diabetes and CV diseases [27,28,29]. The alternate Mediterranean diet (aMed) score is a modified version of an existing Mediterranean diet scale containing nine components including vegetables (not potatoes), fruits, nuts, whole grains, legumes, fish, the ratio of monounsaturated to saturated fat, red and processed meats and alcohol [30]. Greater adherence to a Mediterranean dietary pattern, as measured by the aMed score, did not affect the risk of developing RA or SLE [15] in healthy individuals.

The Dietary Approach to Stop Hypertension (DASH) is a healthy dietary pattern focused on eight components: high intake of fruits, vegetables, nuts and legumes, low-fat dairy products and whole grains, and low intake of sodium, sweetened beverages and red and processed meats [31]. Although previously evaluated only in metabolic diseases, including gout, the recent evidence of a pivotal role of sodium in the induction of autoimmunity led investigators to also explore this pattern in RA and SLE; however no direct association with the risk of developing either disease was demonstrated [15].

The empirical dietary inflammatory pattern (EDIP) includes different foods based on their association with plasma levels of inflammatory biomarkers (including C-reactive protein and IL-6) [32]. The nine anti-inflammatory groups include beer, wine, tea, coffee, dark yellow vegetables, leafy green vegetables, snacks, fruit juice and pizza. The nine pro-inflammatory groups include processed meat, red meat, organ meat, non-dark meat fish, other vegetables, refined grains, high-energy beverages, low-energy beverages and tomatoes. In RA, increasing EDIP scores (namely, most inflammatory dietary intake) were associated with increased risk of seropositive RA among women aged 55 years or below [16]. Conversely, no association between EDIP scores and SLE risk was observed [15].

A detailed analysis of the NHS and NHS II cohorts with regard to the intake of antioxidants from foods and supplements was also conducted; however, it ruled out any protective effect of both the range of antioxidant intakes and of the overall antioxidant intake on the risk of developing RA or SLE [33]. Moreover, it was reported that omega-3 fatty acids could prevent the development of RA, improve muscle metabolism and reduce muscle atrophy in subjects at high metabolic risk [34].

The existing literature does not allow any definitive conclusions to be drawn either in favor or against a role of diet in the risk of developing SLE onset. However, with the only exception of alcohol consumption, studies on other nutrients as well as on dietary patterns are still too few. Therefore, any association (or lack thereof) deserves to be further explored, focusing also, for example, on the possible interaction between food and microbiome, given its role in SLE pathogenesis [35].

## 3. Diet in Established SLE

As mentioned above, diet may have a beneficial role in patients with RMDs such as SLE due not only to a direct action of nutrients on the immune system and inflammation but also to an indirect effect on insulin resistance, obesity and associated co-morbidities [9,10]. However, the existing controversy on diet as a preventive measure for SLE also exists with regard to the role of nutrients as modulators of SLE pathogenesis in patients with overt disease (Table 2). While several studies have been published in experimental SLE, few are available in humans. Furthermore, one main pitfall in investigating diet manipulation not only in SLE but also in other RMDs and chronic diseases is the objective difficulty of setting up an appropriate control group in studies assessing restrictive diets. Therefore, not only are studies scarce but the few available data have a low level of evidence. Finally, yet importantly, studies exploring the role of individual nutrients instead of dietary patterns may provide a biased picture. In fact, single dietary components may either confer only modest benefits or behave differently following the interaction with other nutrients within the more complex scenario of dietary patterns.

### 3.1. Studies on Experimental SLE

Studies that evaluated the role of diet in experimental SLE shed some light on the modulation of the immune system by various nutrients. A low-calorie diet was able to impact on the cellular and humoral immune responses by reducing the titers of anti-double-stranded DNA antibodies [47], B:T-cell and CD4+:CD8+ T-cell ratios [48], and interferon gamma [49] in the lupus-prone mouse model (NZB/NZW F1). With regard to intermittent fasting, while one study reported an increase of regulatory T lymphocytes in (NZB × NZW) F1 lupus-prone mice [50], another described an exacerbation of lupus nephritis in MRL/lpr lupus-prone mice by increasing the survival and autophagy in antibody-producing B lymphocytes [51].

As far as PUFAs are concerned, an isocaloric diet containing docosahexaenoic acid (DHA) reduced cytokine and chemokine synthesis, lymphocyte infiltration and autoantibody synthesis, ultimately preventing the occurrence of glomerulonephritis in NZBWF1 lupus-prone female mice [52,53]. Likewise, the intake of PUFA-rich extra-virgin olive oil reduced disease activity in a pristane-induced SLE mouse model not only by modulating proinflammatory cytokines but also by regulating prostaglandin E2 levels in the kidney and matrix metalloproteinase-3 in the serum [54]. In addition, extra-virgin olive oil and its phenol fraction proved able to counteract inflammatory pathways in cells of the monocyte–macrophage lineage in a pristane-induced BALB/c mouse model with SLE [55].

Polyphenols present in green tea, such as epigallocatechin-3-gallate (EGCG), also showed a possible activity against the pro-inflammatory status in NZB/W F1 SLE-prone mice. EGCG was able to both reduce renal inflammasome activation and boost antioxidant signaling pathways [56].

Finally, data on curcumin, one of the turmeric polyphenols, are conflicting since despite exerting a protective effect against lupus nephritis [57], it was found also able to worsen SLE neurological involvement in SLE-prone MRL/lpr mice [58].

### 3.2. Human Studies

As mentioned above, the Mediterranean diet is a dietary pattern enriched in fiber and olive oil, the latter being a source of mono-unsaturated fatty acids; it is also characterized by a high intake in fish, an important source of dietary n-3 polyunsaturated fatty acids (PUFAs) [59]. A meta-analysis of randomized controlled trials (RCTs) assessing the effect of n-3 PUFA supplementation in RA showed a small, albeit significant, improvement of joint pain, acute phase reactants and physical function [36]. In addition, a study reported that monounsaturated fatty acids (MUFAs), as part of a Mediterranean diet, were linked with disease activity in RA since high MUFA intake was an independent predictor of remission, and the ratio of daily consumption of MUFA to saturated fatty acids (MUFAs/SFAs) was inversely correlated with disease activity [60].

Although some results suggest omega-3 fatty acids are able to improve SLE conditions by positively modulating some inflammatory pathways, some research has indicated ambiguity concerning their effectiveness in SLE [61]. A short-term (12 weeks) RCT failed to observed any benefit on disease activity or endothelial function (measured by flow-mediated dilation (FMD) of the brachial artery) [41], while a significant improvement of both was observed at both 12 and 24 weeks in another RCT [42]. These differences may be at least partly explained by the results of a meta-analysis of RCTs assessing the effects of PUFAs on endothelial function in normal individuals. The authors reported an improvement of FMD occurring at a median of 56 days, but this effect could be modified according to the health status of the participants and the dose of supplementation [62]. In both these RCTs, the regimen was 1.8 g eicosapentaenoic acid (EPA) and 1.2 g docosahexaenoic acid (DHA); therefore, other factors likely related to the characteristics of the patient cohort could explain this discrepancy.

Two other RCTs described an improvement of patient global assessment at 24 [63] and 34 weeks [64]. Finally, two RCTs reported no effect of PUFA supplementation on serum levels of IL-6 and IL-10 [43] or IL-12 and IL-13 [63]. It is worth mentioning that an increase of low-density lipoprotein (LDL) was reported in one RCT at 12 weeks [41].

The only study exploring the effects of adherence to the Mediterranean diet in SLE, a cross-sectional study conducted in 280 patients and using a validated questionnaire, revealed that patients adhering the most to this dietary pattern showed not only lower disease activity and disease damage scores but also lower body mass index, lower fat mass and fewer CV disease risk factors. Furthermore, greater consumption of olive oil, fruits, vegetables and fish while abstaining from red meat and meat products and sugars was associated with lower disease activity and damage [44]. These results are reinforced by those of an observational study exploring the dietary habits of patients with SLE [45]. The authors reported that despite a global energy intake comparable to that of healthy controls, patients with SLE had lower fiber and PUFA intake, and an inverse relationship between PUFA levels and disease activity was observed. Of note, SLE patients with a less healthy dietary pattern also displayed more severe subclinical atherosclerosis as measured via the intima-media thickness on carotid ultrasonography [45]. In addition, overweight and obesity are rather frequent in SLE, even in subjects apparently well-nourished based on their dietary patterns [65]; obesity is an independent risk factor for higher disease activity [66].

While the exact mechanisms underlying the possible positive effects of the Mediterranean diet on the immune system remain to be fully elucidated, the benefit resulting from the metabolic improvement is otherwise rather clear. Therefore, patients with SLE should be directed to nutritional counselling in order to adapt their dietary pattern towards the Mediterranean diet.

Furthermore, albeit flavonoids, the most abundant phenolic compounds with anti-oxidant, anti-inflammatory and immunomodulatory properties represented in Mediterranean diet [67], seem to be implicated in most benefits to control inflammation and alleviate arthritis symptoms in human and experimental models of RA [68], their specific role in the development and influence in the inflammatory mechanism of RA as well as SLE has yet to be clarified [69]. Of interest, however, a recent study revealed a direct association of fruit-derived polyphenols and specific intestinal microorganisms in SLE, setting the stage for further research on diet and the microbiome [70]. Intestinal dysbiosis seems to be yet another facet of SLE pathogenesis [35], and therefore it is intriguing to hypothesize that dietary counselling in this disease may lead to a modulation of the microbiome and ultimately to a restoration of a normal gut flora.

As far as restriction diets are concerned, two open studies on gluten-free and vegan diet suggested a slight improvement of disease activity in RA [37,38]. However, no data are available in SLE. It should be noted, however, that studies on people with or without coeliac disease and those on gluten-free diets warned of an increased CV risk [71,72]. Since patients with RMDs are burdened by a higher CV risk compared to the general population, the choice of a gluten-free diet should be avoided, unless required by a concomitant coeliac disease.

Data on a lactose-free diet is lacking in RA and SLE while several open studies assess fasting protocols in RA [39,40]. Although providing a certain benefit on pain, morning stiffness and inflammation, fasting should be considered with caution since a rebound upon food resumption was observed. Only one study assessed the effects of fasting, in particular Ramadan fasting, in SLE by enrolling a small cohort of patients with quiescent disease [46]. After an average of 24 days of fasting, a significant increase of anti-dsDNA antibodies was observed compared to non-fasting controls. This increase remained stable and significant after three months. However, no changes in disease activity and patient-reported quality of life (as measured by the short form-36 questionnaire) were observed. At three months, total cholesterol was significantly lower in fasting versus non-fasting patients.

Finally, the metabolic effect of some treatments, in particular glucocorticoids (GCs), in RA and SLE needs to be appraised. Long-term use of GCs is associated with arterial hypertension, hyperglycemia, obesity and hyperlipidemia (for which conflicting evidence exists). Unfortunately, although providing some dietary advice for patients treated with GCs, such as reducing salt, sugar, and caloric intake while increasing the intake of protein and potassium, no official recommendations have been released so far [73].

## 4. Conclusions

There is less evidence on diet and SLE in comparison to that on diet and RA, and studies on dietary patterns have failed to identify any relevant association with the risk of developing SLE in normal subjects. Conversely, a reduced risk of RA related to a healthier diet and an increased risk in relation to a more inflammatory dietary intake have been reported. Studies on experimental SLE and on patients with established disease suggest a positive role for some nutrients, such as PUFAs and moderate alcohol intake, in line with what is observed in RA. However, although we acknowledge that similarities exist between the two diseases, at the moment evidence is stronger in RA; therefore, additional studies should be conducted in SLE to shed additional light on this topic.

Nonetheless, given the positive effects on the metabolic profile and CV risk, the Mediterranean diet may be suggested as the preferred dietary pattern in SLE, and nutritional counselling should be part of the multidisciplinary approach in patients with SLE to prevent obesity, malnutrition and the use of food that may fuel an inflammatory status and CV risk.

## Figures and Tables

**Table 1:** Comparison of data available about different dietary patterns and the risk of developing rheumatoid arthritis (RA) and systemic lupus erythematosus (SLE) in normal subjects.

Dietary Pattern or Nutrient, Ref	N	RA	N	SLE
AHEI-2010 total score [14,15]	76,597	↓ risk	204,055	no association
AHEI-2010 alcohol domain (moderate intake) [14,15]	76,597	↓ risk	204,055	no association
AHEI-2010 red meat domain (lower intake) [14,15]	76,597	↓ risk	204,055	no association
AHEI-2010 nut/legume intake (higher intake) [14,15]	76,597	no association	204,055	↓ risk
aMed [15]	204,055	no association	204,055	no association
DASH [15]	204,055	no association	204,055	no association
EDIP [15,16]	173,560	↑ risk	204,055	no association

AHEI, alternative healthy eating index; aMed, alternate mediterranean diet; DASH, dietary approach to stop hypertension; EDIP, empirical dietary inflammatory pattern.

**Table 2:** Comparison of the effects (or lack thereof) of nutrients/dietary patterns in patients with established rheumatoid arthritis or systemic lupus erythematosus.

	Author, Ref	Dietary Pattern or Nutrient	Study Type	Follow Up	N Patients		Results
					Intervention	Control	
RA	Gioxari et al. [36]	Mediterranean diet/PUFAs	Meta-analysis of RCTs	NA	NA	NA	Improvement of joint pain, acute phase reactants and physical function
	Elkan et al. [37]	Gluten-free and vegan diet *	RCT	12 m	38	28	Improvement of disease activity
	Hafstrom et al. [38]	Gluten-free and vegan diet *	RCT	12 m	38	28	Improvement of disease activity
	Muller et al. [39]	Fasting	Systematic literature review	NA	NA	NA	Improvement of pain, morning stiffness and inflammation
	Uden et al. [40]	Fasting	Longitudinal	3–6 w	4	9	Improvement of pain, morning stiffness and inflammation
SLE	Bello et al. [41]	PUFAs	RCT	12 w	42	45	No effect on disease activity No improvement of endothelial function
	Wright et al. [42]	PUFAs	RCT	24 w	30	30	Improvement of disease activity; improvement of endothelial function
	Curado Borges et al. [43]	PUFAs	RCT	12 w	22	27	No effect on serum levels of IL-6 and IL-10
	Pocovi-Gerardino et al. [44]	Mediterranean diet	Cross sectional, non-interventional	NA	SLE N = 280		Improvement of disease activity
	Elkan et al. [45]	Mediterranean diet	Cross sectional, non-interventional	NA	SLE N = 114 NC N = 122		Improvement of disease activity Improvement of endothelial function
	Goharifar et al. [46]	Fasting	Longitudinal	24 ± 5 d	21	19	No effect on disease activity

* no data available on systemic lupus erythematosus; d = days; w = weeks; m = months; NA = not applicable; NC = normal controls; PUFAs = polyunsaturated fatty acids; RCT = randomized controlled trial; SLE= systemic lupus erythematosus.
